# Positioning of epidural electrode for motor cortex stimulation in general anesthesia based on intraoperative electrophysiological monitoring to treat refractory trigeminal neuropathic pain

**DOI:** 10.1007/s00701-023-05801-5

**Published:** 2023-09-15

**Authors:** Vesna Malinova, Tammam Abboud, Veit Rohde, Dorothee Mielke

**Affiliations:** Department of Neurosurgery, University Medical Center Göttingen, Georg-August-University, Robert-Koch-Straße 40, 37075 Göttingen, Germany

**Keywords:** Motor cortex stimulation, Neuropathic pain, Neuromodulation

## Abstract

**Background:**

Motor cortex stimulation (MCS) represents a treatment option for refractory trigeminal neuralgia (TGN). Usually, patients need to be awake during surgery to confirm a correct position of the epidural electrode above the motor cortex, reducing patient’s comfort.

**Method:**

Epidural cortical mapping (ECM) and motor evoked potentials (MEPs) were intraoperatively performed for correct localization of motor cortex under general anesthesia that provided comparable results to test stimulation after letting the patient to be awake during the operation.

**Conclusion:**

Intraoperative ECM and MEPs facilitate a confirmation of correct MCS-electrode position above the motor cortex allowing the MCS-procedure to be performed under general anesthesia.

**Supplementary Information:**

The online version contains supplementary material available at 10.1007/s00701-023-05801-5.

## Background

Trigeminal neuralgia (TGN) manifests with severe pain attacks along the trigeminal nerve distribution, representing one of the most common causes of facial pain. Although approximately 70–90% of affected patients experience a satisfactory pain relief after the initiation of pharmacotherapy, approximately 44% of TGN patients suffer from refractory pain attacks from a long-term point of view [1]. Surgical techniques, including microvascular decompression (MVD), percutaneous rhizotomy or stereotactic radiosurgery, should be considered as valuable treatment options for TGN refractory to medical treatment. Refractory TGN is a challenging condition due to a limited number of effective therapeutic options. Consequently, the continuous development of neuromodulation techniques represents a promising salvage treatment option in case of refractory neuropathic pain. In the early 1990s, motor cortex stimulation (MCS) has been proven to be effective in reducing TGN attacks not sufficiently controlled by medical treatment and common surgical procedures [6]. Since then, several case series have been published with a reported median pain reduction of 70%, based on the pre- and post-operative visual analog scale, as stated in a recently published review [3]. The correct position of the epidural electrode above the motor cortex is the major prerequisite for successful treatment. Hence, the procedure is preferentially performed under local anesthesia in order to conduct an intraoperative test-stimulation, confirming the correct electrode position [2, 4, 9]. Additionally, intraoperative electrophysiological monitoring and neuronavigation are being implemented for the localization of the central sulcus. Median nerve somatosensory evoked potential (SEP) is usually used to identify the position of the central sulcus by recording a N20/P20 phase reversal across the central sulcus [5]. Unfortunately, awake surgical procedures are generally associated with discomfort for the patients. In order to address the need for a more comfortable alternative, we present an alternative surgical option to confirm the correct position of the electrode above the motor cortex by intraoperatively eliciting motor evoked potentials (MEPs) after direct cortical stimulation via the placed epidural electrode under general anesthesia.

## Method

### Relevant surgical anatomy and technique description

**Fig. 1:**
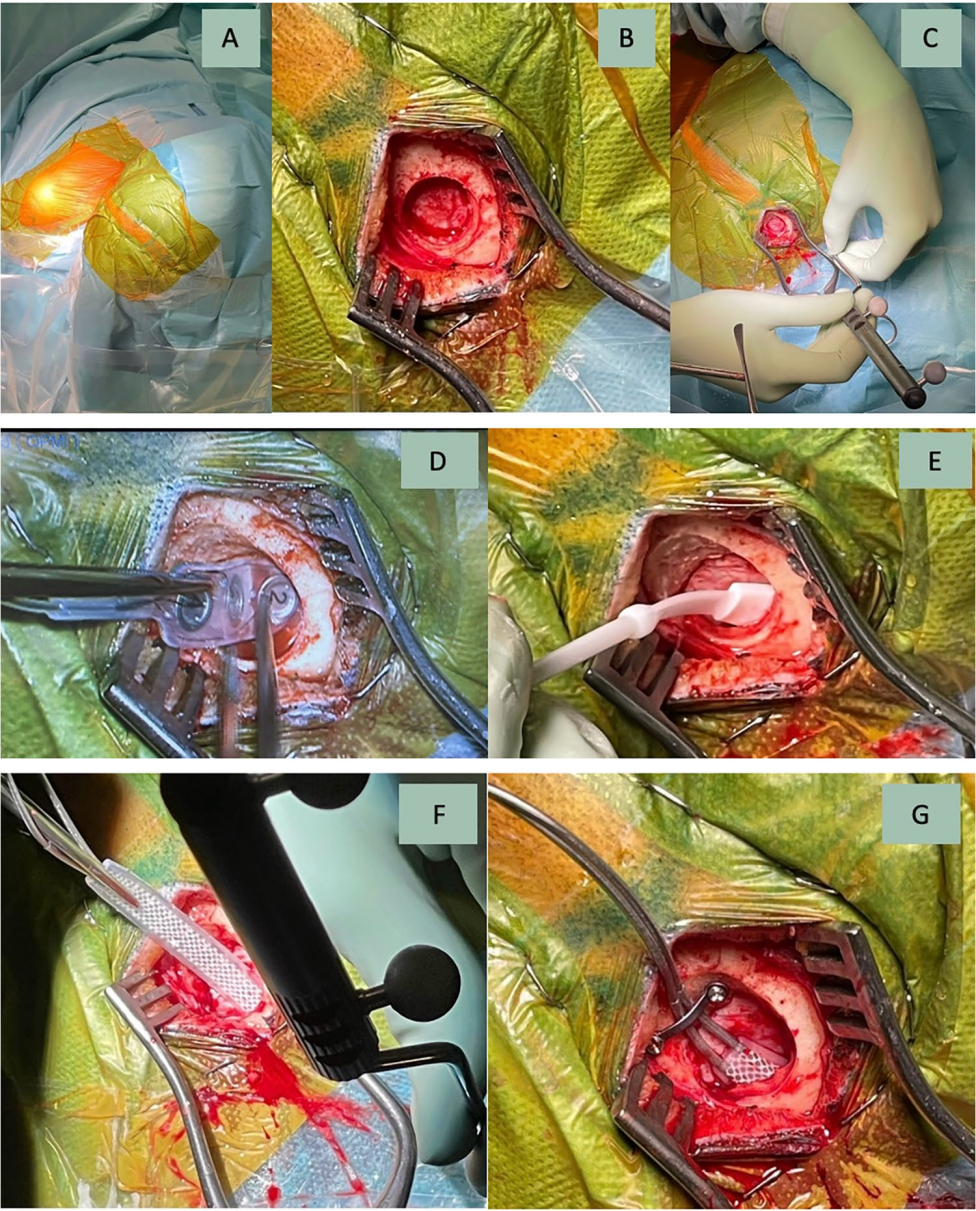
Intraoperative setup chronologically demonstrating the individual operative steps: **A** Depiction of the operative field after sterile coverage. **B** Depiction of the situs after a skin incision (2.5 cm length) and a burr hole (2 cm laterally from the midline, with 1 cm diameter) were performed, with a self-retractor in situ. **C** Localization of the insertion direction of the electrode along the motor cortex by means of the navigation pointer. **D** Epidural insertion of a four-contact grid electrode crossing the central sulcus with subsequent demonstration of a phase inversion as a confirmation of the correct localization of the central sulcus. **E** Epidural insertion of the electrode phantom for separation of the dura from the bone. **F** Epidural insertion of the electrode guided by the navigation pointer. **G** Depiction of the positioned epidural electrode with fixation using titanium miniplates on the burr hole edge

**Fig. 2:**
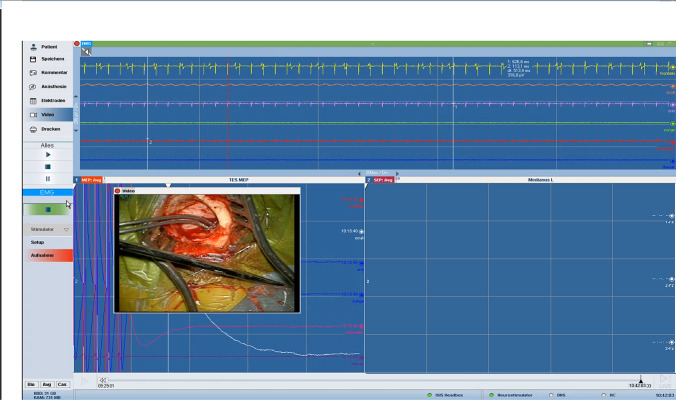
A screen shot from intraoperative electrophysiological monitoring showing muscle action potentials recorded from two facial muscles (orbicularis oris and frontalis) after monopolar epidural stimulation with a strip electrode using a current intensity of 7 mA

**Fig. 3:**
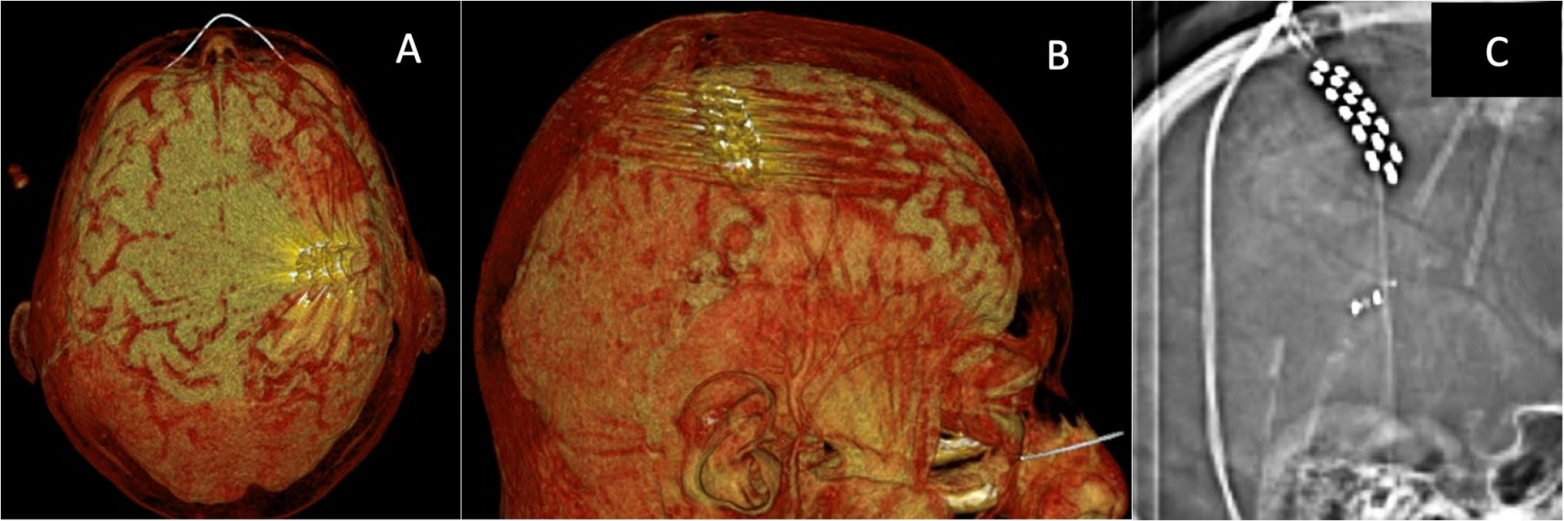
Postoperative electrode position in the computed tomography (CT) scan. **A** 3D reconstruction of the CT data set (syngo.via software, Siemens Healthinears) with depiction of the electrode positioned over the right motor cortex from a superior-inferior perspective. **B** 3D reconstruction of the CT data set (syngo.via software, Siemens Healthinears) with depiction of the electrode positioned over the right motor cortex from a lateral perspective. **C** Localizer (topogram) from the CT data set with depiction of the implanted electrode encompassing 16 stimulation contacts

The correct identification of the central sulcus is an essential prerequisite for the reliable electrode positioning and, thereby, for a successful treatment. The central sulcus is localized using the preoperatively performed magnetic resonance imaging (MRI). A functional MRI (fMRI) may assist in identifying the motor cortex. The preoperative planning can be deployed intraoperatively to identify the central sulcus and for localizing the electrode entry point as well as the electrodes’ alignment. After induction of general anesthesia (consisting of remifentanil with a dosage of 0.3 μg/kg/min and propofol with a dosage of 6 mg/kg/h), the patient is positioned in a supine position. The patient’s head is fixed in a Mayfield clamp and rotated to the affected painful side. The navigation dataset, containing the preoperatively segmented motor cortex area by means of the Brainlab software Elements using object creation and supported by fMRI, is registered and the planned skin incision is marked. The skin incision has a length of approximately 2.5 cm and is placed parallel to the localized central sulcus. Electromyography electrodes are placed in appropriate muscle groups to monitor muscle contractions of the contralateral face and arm. A burr hole is placed approximately 2 cm paramedian and the dura is separated from the bone with a blunt dissector. Afterwards, a grid electrode is inserted epidurally, crossing the central sulcus, until detecting a phase reversal, confirming the correct localization above the central sulcus. The epidural electrode (Specify 5-6-5 Medtronic was used here) is pushed forward into the direction of the distal motor cortex, guided by navigation until all contacts disappear under the bone edge of the burr hole. Afterwards, MEPs are recorded through the positioned epidural electrode. By activating different contacts, an electromyogram of the affected muscles is generated and information is being gathered concerning the electrode position in relation to the motor cortex. After electrophysiologically confirming the correct electrode position, the two electrode wires are connected to an extension wire, which is externalized in the region of the shoulder girdle after subcutaneous tunneling. The intraoperative measurements are being verified after extubation of the patient followed by a dedicated adjustment of different stimulation programs. The trial period usually comprises approximately 1 week. The intraoperative setup is step-by-step demonstrated in Fig. 1. A screen shot from intraoperative electrophysiological monitoring showing MEP recorded from two facial muscles (orbicularis oris and frontalis muscles) after monopolar epidural stimulation with a strip electrode using a current intensity of 7 mA is given in Fig. 2. Postoperatively, a computed tomography (CT) scan is conducted with 3D reconstruction of the data set with depiction of the positioned electrode, confirming the correct position over the motor cortex (Fig. 3). A video showing the intraoperative electrophysiological evaluation with cortical SEP confirmation of the precentral position of the strip electrode and with the stimulation results of monopolar stimulation with the 16-contact electrode was uploaded with the manuscript (Video 1).

## Indications

Since MCS is still an off-label procedure, it is indicated as an individual therapeutic attempt in patients with TGN refractory to medical and other established surgical treatments.

## Limitations

Patients with a previous craniotomy in the region of the central sulcus might exhibit epidural scarring. The exact localization of the position of central sulcus may be challenging in some cases, which can limit the success of the procedure.

## How to avoid complications

Possible complications of MCS are hemorrhage (2.5%), infection (2.2–5.7%), transient neurological deficits (2.5%), hardware-related complications (5.1%), as well as stimulation-related complications like seizures (12%) [7, 8]. A meticulous separation of the dura from the bone should be carried out before placing the electrode. Pushing the electrode against resistance should be avoided. We propose to fix the electrode on the skull with a small titanium plate in order to avoid a dislocation during the tunneling maneuver of the cable.

## Specific perioperative considerations

It is important to select appropriate patients for a neuromodulation procedure and to communicate realistic expectations for the alleviation of pain attacks. A meticulous preoperative planning with segmentation of the motor cortex (3D T1 MRI dataset) is indicated to facilitate the intraoperative neuronavigation guidance of the procedure.

## Specific information to give to the patient about surgery and potential risks

The main risks of the procedure are hemorrhage and infection. Other possible risks are dura perforation with motor cortex injury and cerebrospinal fluid leakage. The patients have to be informed about the risk of electrode dislocation with subsequent loss of stimulation effects and the possible need for revision surgery. Although the majority of patients show a response to MCS [5], in case of non-response to MCS, the electrode has to be removed in another surgical procedure.

## Summary

We present a method to perform MCS-procedure under general anesthesia. After, neuronavigational-guided electrode placement with confirmation of electrode position over the motor cortex by epidural cortical mapping of the motor cortex and MEPs makes awake surgery superfluous in this patient cohort.

### Supplementary information

Supplementary file 1 **Video 1** Intraoperative electrophysiological evaluation with cortical SEP confirmation of the precentral position of the strip electrode and with the stimulation results of monopolar stimulation with the 16-contact electrode. (MOV 59319 kb)

## Data Availability

All available data was presented in the manuscript.
